# Highly synergistic drug combination prevents vaginal HIV infection in humanized mice

**DOI:** 10.1038/s41598-020-69937-5

**Published:** 2020-08-03

**Authors:** Marc M. Baum, Christina M. Ramirez, John A. Moss, Manjula Gunawardana, Michael Bobardt, Philippe A. Gallay

**Affiliations:** 1 Department of Chemistry, Oak Crest Institute of Science, 128-132 W. Chestnut Ave., Monrovia, CA USA; 2 Los Angeles (UCLA) Fielding School of Public Health, University of California, 650 Charles E. Young Dr. South, 16-035 Center for Health Sciences, Los Angeles, CA USA; 3 Department of Immunology and Microbiology, The Scripps Research Institute, 10550 North Torrey Pines Road, La Jolla, CA USA

**Keywords:** Experimental models of disease, HIV infections

## Abstract

The HIV-1 epidemic remains an urgent global health concern. Young women are disproportionately at risk of acquiring the virus. A range of highly effective, female-controlled, discrete vaginal products therefore is needed to help curb the epidemic. Oral tenofovir disoproxil fumarate (TDF) and emtricitabine (FTC) are effective in HIV-1 pre-exposure prophylaxis (PrEP) and form a promising basis for a vaginal product. Here, we evaluate TDF and FTC in combination with the broadly neutralizing antibody VRC01-*N* using a highly reproducible humanized mouse model. The agents were vaginally dosed individually and in combination, and the efficacy of HIV-1 prevention was analyzed using the established, rigorous median-effect model. Surprisingly, the triple combination showed a high degree of synergism, unprecedented for in vivo HIV-1 PrEP, leading to a possible fivefold dose reduction for some of the agents. Vaginal administration of the TDF-FTC-VRC01-*N* combination holds significant promise for HIV-1 PrEP.

## Introduction

The statistics surrounding the global HIV-1 epidemic remain alarming, despite significant treatment and prevention efforts. As of late 2018, 74.9 million people have become infected since the start of the pandemic, and 32.0 million people have died from AIDS-related illnesses^1^. The global, annual infection rate of 1.7 million people^1^ indicates that a prevention gap has been reached^2^, likely hampering our ability to meet the aggressive Fast-Track goals set by the Joint United Nations Programme on HIV/AIDS (UNAIDS). These include 500,000 (or fewer) new annual infections by 2020 and an end to the AIDS epidemic by 2030^3^.

A recent, randomized, multicenter, open-label clinical trial across 12 research sites in Eswatini, Kenya, South Africa, and Zambia involving HIV-1 seronegative women aged 16–35 years unexpectedly found highly concerning HIV-1 incidence rates of 4 per 100 woman-years^4^. Young women (ages 15–24) are disproportionately at risk, with an estimated 6,000 new HIV-1 infections occurring weekly^1^, and are twice as likely to be living with HIV-1 than men. In sub-Saharan Africa, four in five new infections are among adolescent girls aged 15–19 years^1^. To meet the ambitious UNAIDS targets, highly effective biomedical modalities for HIV-1 prevention in young women are urgently needed.

Pre-exposure prophylaxis (PrEP) against HIV-1 using an oral regimen of the antiretroviral (ARV) agents tenofovir disoproxil fumarate (TDF) and emtricitabine (FTC) has been initiated in numerous countries around the world^5^, but discontinuation is high in multiple populations^6–9^. The future success of HIV-1 PrEP depends on a broad range of product choices being available to end-users. For young women in sub-Saharan Africa, female-controlled, discrete, on-demand vaginal products, such as gels, films, tablets, and intravaginal rings (IVRs) have favorable end-use characteristics^10^. These topical modalities also have the potential advantage of limited systemic drug absorption and no apparent drug resistance in breakthrough infections.

An optimally effective HIV-1 PrEP strategy likely will require multiple ARV agents, by analogy to highly active antiretroviral therapy (HAART)^11^, and optimal combinations will need to be determined in systematic studies using in vitro single-round HIV-1 infectivity assays^12^. Building on the clinical success of TDF-FTC in HIV-1 PrEP^13–20^, we selected the broadly neutralizing antibody (bNAb) VRC01 as a complementary third agent for evaluation. Systemic and topical VRC01 is currently undergoing clinical testing as an agent for HIV-1 PrEP^21^, and prevents infection using a different mode of action from the two small-molecule ARV agents. The antibody prevents viral entry by partially mimicking the interaction between the host CD4 receptor and the HIV-1 gp120 envelope glycoprotein^22^ and is capable of neutralizing 91% of known HIV-1 isolates in vitro^23^. The viability of complex drug combinations needs to be investigated using a rational, data-driven approach.

Herein, we describe the use of humanized bone marrow/liver/thymus (BLT) mice to evaluate the efficacy of anti-HIV-1 agents, individually and in combination, in preventing vaginal HIV-1 acquisition. The empirical Chou–Talalay model^24,25^ is used to quantitatively study dose–effect relationships, leading to the identification of strong synergy in a novel three-drug combination consisting of TDF, FTC, and VRC01-*N*, where “*N*” designates bNAb produced in transiently and stably transformed *Nicotiana* spp.^26^.

## Results

### Characteristics of VRC01-*N*

The molecular weight of the VRC01-*N* produced in *Nicotiana* spp. used here was measured as 157 kDa, based on reduced SDS-PAGE gels (Fig. S1 in Supplementary Information; heavy chain, 49.5 kDa; light chain, 28.8 kDa), and reduced and non-reduced gel data reported by Teh et al.^26^ The above value is in agreement with the predicted molecular weight based on amino acid sequence analysis of the heavy and light chains of the dimeric monoclonal antibody (mAb) molecule^26^. This molecular weight was used to convert mAb mass concentrations to molar concentrations.

The HIV-1 gp120 binding activity of the VRC01-*N* used in the current study was compared to the reference material (produced in a HEK 293-6E expression system) obtained from the National Institutes of Health (NIH) AIDS Reagent Program (aidsreagent.org) using an enzyme-linked immunosorbent assay (ELISA). The VRC01-*N* activity (slope 0.1462, OD_450_ against [VRC01], in µg mL^−1^) was 16.6 times higher than that of the reference material (slope 0.008817, OD_450_ against [VRC01], in µg mL^−1^) based on this assay, and a comparison of the linear regression of optical density at 450 nm versus concentration (0.08–10 µg mL^−1^).

### BLT mouse study design and efficacy endpoints

The degree of humanization of the BLT mice was verified at 20 weeks of age, 10 weeks post-CD34^+^ hematopoietic stem/progenitor cell (HSPC) injection, prior to each challenge study by collecting peripheral blood and analyzing it by fluorescence-activated cell sorting (FACS) for percentages of human CD45^+^ cells and human CD45^+^ CD4^+^ CD3^+^ cells. These data are included in the Supplementary Information for each infectivity study. Mice that did not exhibit sufficient percentages of human cells (< 65% of CD45^+^ cells and < 70% of CD3^+^ and CD4^+^ cells) were not used in infection studies.

**Figure 1:**
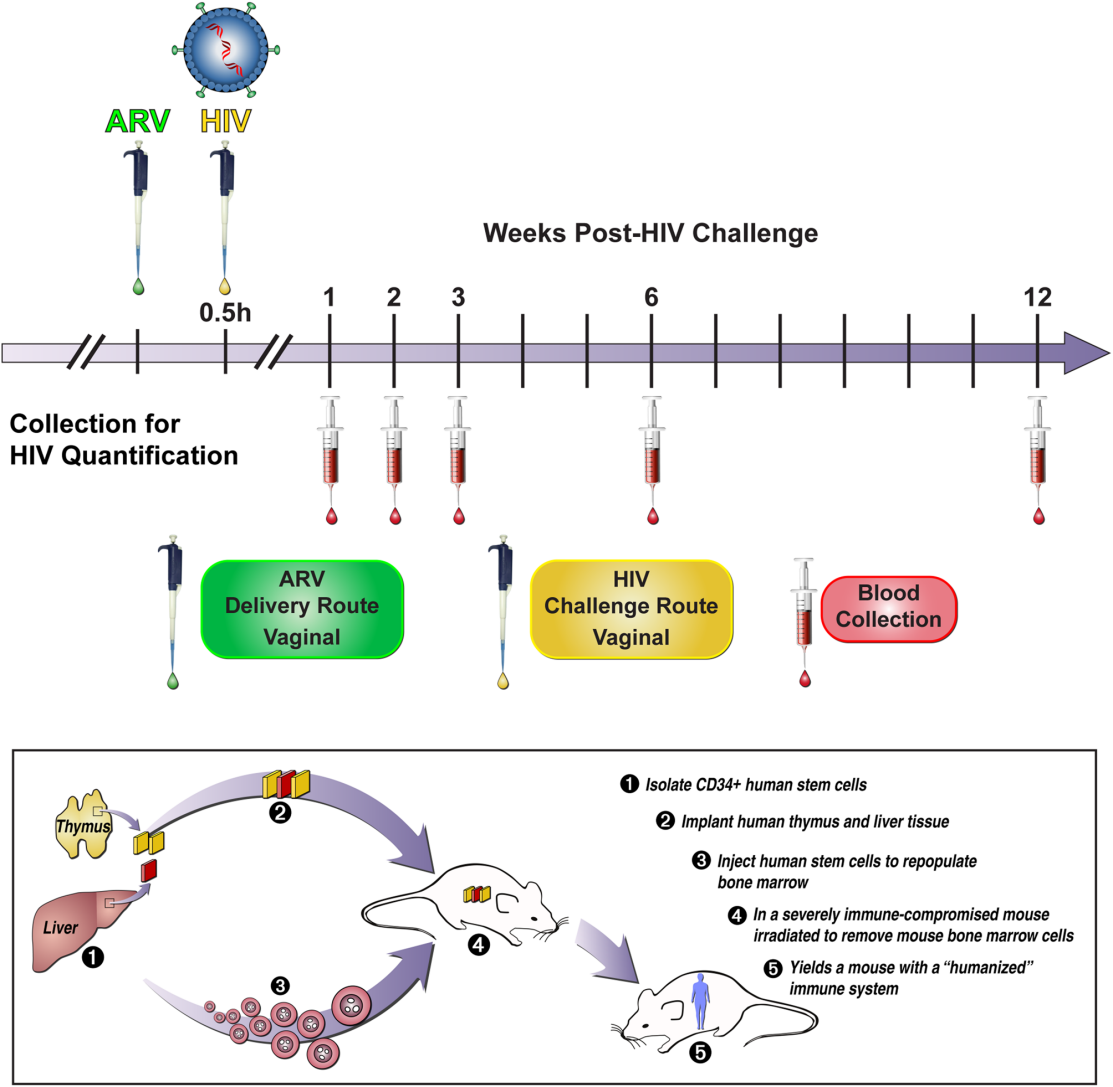
Experimental design of humanized BLT mouse HIV-1 PrEP studies. Vaginal application of drug solution in PBS (green box) was followed by the HIV-1 exposure (yellow box) within 30 min (typically 10 ± 15 min). Peripheral blood samples were collected at the indicated times (red box) and HIV-1 viral load measured by qPCR.

After confirming the reconstitution of mice with human cells, a series of exploratory vaginal HIV-1 challenge studies over a broad dose range were conducted according to the schematic shown in Fig. 1. These preliminary studies allowed approximate median effective doses (ED_50_) to be determined and were followed up with additional studies over a narrower dosing range. In all cases where mice were HIV-1 infected, viral RNA was detected at 1, 2, 3, 6, and 12 weeks post challenge: TDF, 1.37 ± 0.38 × 10^5^ copies mL^−1^; FTC, 1.41 ± 0.41 × 10^5^ copies mL^−1^; VRC01-*N*, 1.41 ± 0.45 × 10^5^  copies mL^−1^ (Appendix A in Supplementary Information); TDF-FTC-VRC01-*N*, 1.54 ± 0.65 × 10^5^ copies mL^−1^ (Appendices B and C in Supplementary Information). The magnitude and consistency of these values support the robustness of the experimental model. When the mice were protected from HIV-1 infection, no viral RNA was detected at any of these timepoints.

A sample size of ten mice per group yields 80% power at an effective alpha level of 0.038 to detect a difference of 0.46 using a two-sided binomial test. This test assumes the underlying population proportion under the null hypothesis is 0.1. Similarly, a sample size of eight mice per group yields 80% power at an effective alpha level of 0.0354 to detect a difference of 0.54 using a two-sided binomial test. This test assumes the underlying population proportion under the null hypothesis is 0.35.

### Dose–response and slope parameters for single and triple drug combinations

**Figure 2:**
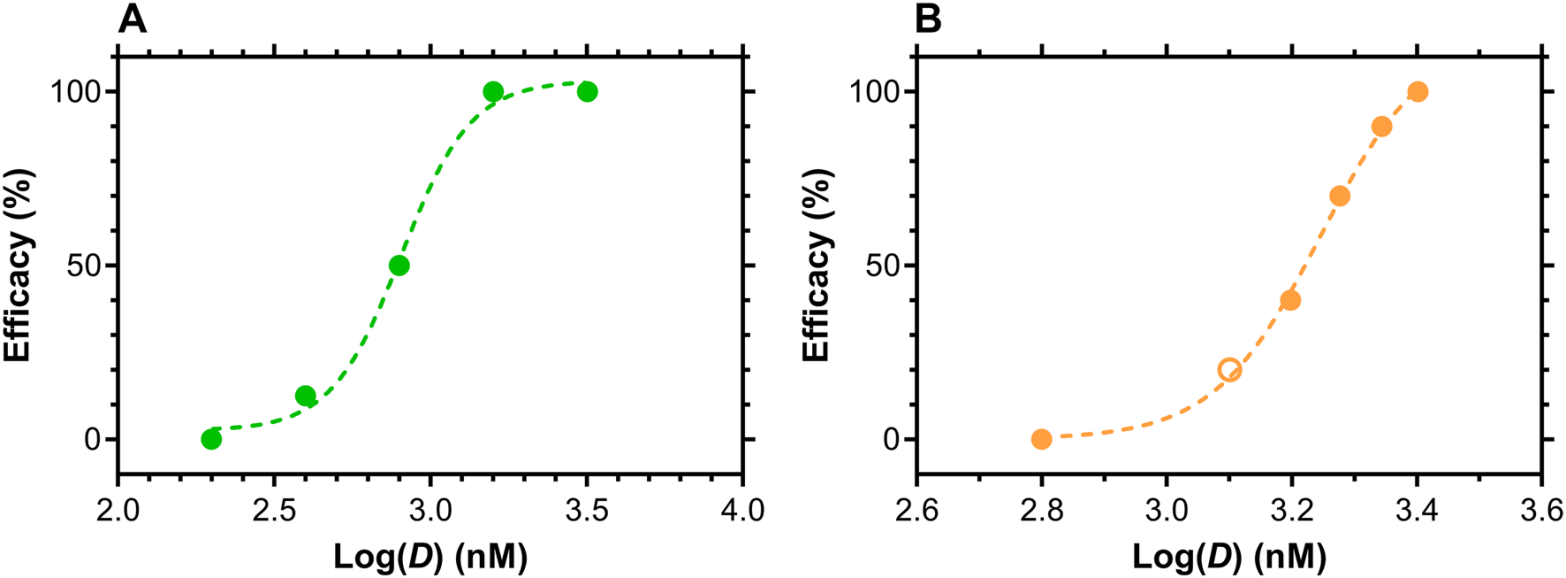
Dose–response curves for vaginal HIV-1 challenge studies in humanized BLT mice. Plots of efficacy *versus* dose of (**A**) VRC01-*N* (*N* = 8 per dosing group, 5 groups) and (**B**) TDF-FTC-VRC01-*N* combination (*N* = 10 per dosing group, 5 groups per study; two studies) applied prior to HIV-1 challenge. Open circle corresponds to datapoint from separate experiment. Dashed lines are fits to a sigmoidal dose–response (variable slope) model used to calculate EC_50_ of the drug or drug combination providing protection against vaginal HIV-1 challenges.

The dose–response relationships for VRC01-*N* (*N* = 8 per dosing group, five groups, Appendix A under Supplementary Information) and TDF-FTC-VRC01-*N* (*N* = 10 per dosing group, five groups, Appendices B and C under Supplementary Information) are presented in Fig. 2. Analogous curves for TDF, FTC, and the TDF-FTC combination have been reported previously^27^.

The EC_50_ values for the single drug systems and the slope parameters (*m*-values, vide infra) are shown in Table 1.

**Table 1:** Dose–response characteristics of vaginally applied agents in BLT mice.

Anti-HIV drug	EC_50_ (µM)	*m*-value, *R*^*2*^
TDF	4.6^a^	3.10, 0.977
FTC	0.56^a^	2.82, 0.966
VRC01-*N*	0.81^b^	4.00, 0.970
TDF-FTC-VRC01-*N*	N/A^c^	5.62, 0.980

^a^See Gallay et al.^27^.

^b^0.13 mg mL^−1^.

^c^See Fig. 3.

### Empirical analysis of drug combination effects on HIV-1 preventative efficacy

**Figure 3:**
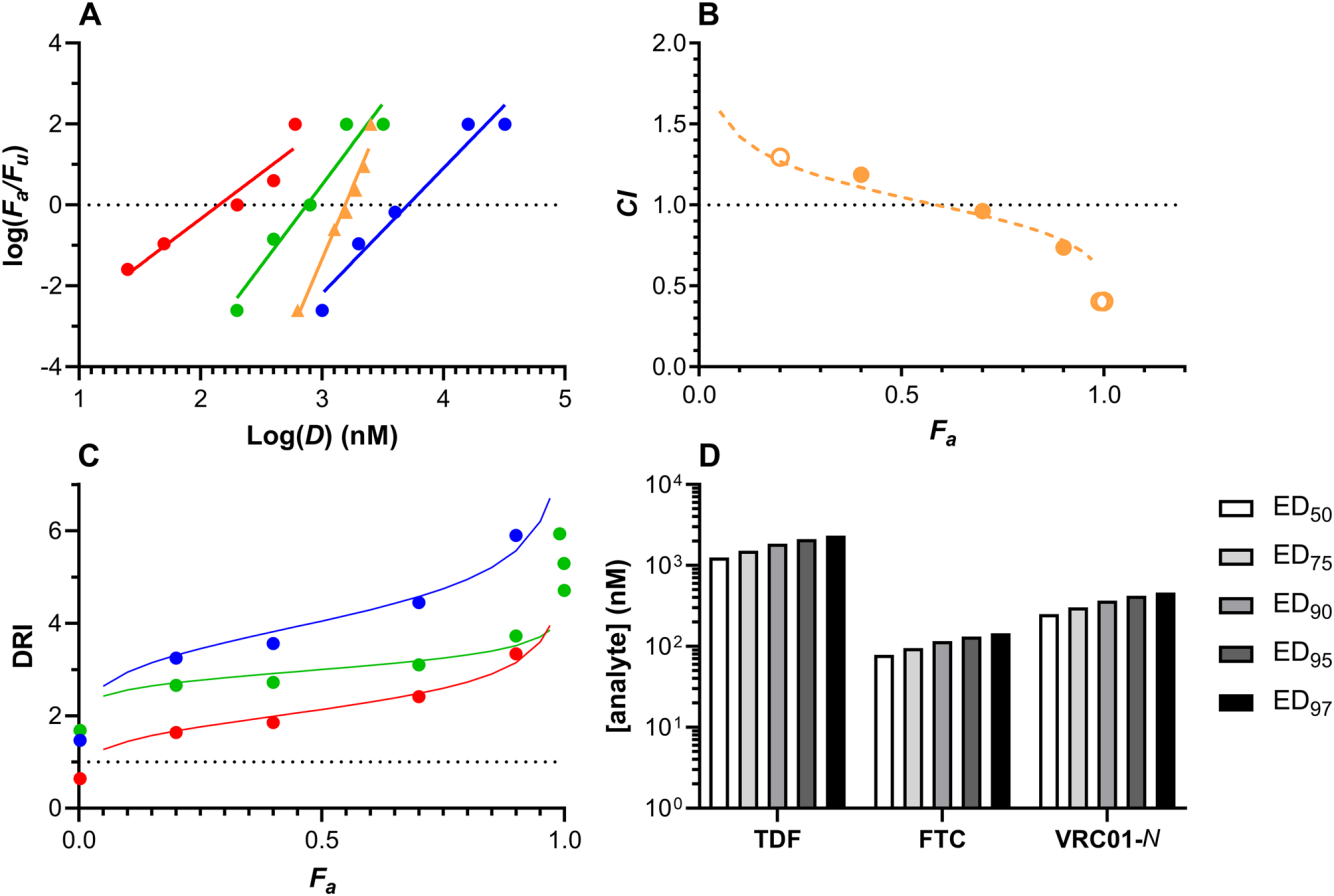
Median-effect model analysis of efficacy in vaginal HIV-1 prevention using a triple drug combination (*N* = 10 per dosing group, 5 groups per study). *F*_*a*_, fraction affected; *F*_*u*_, fraction unaffected; *D*, dose (nM). For panels (**A**–**C**) Blue, TDF; red, FTC, green; VRC01-*N*; orange, TDF-FTC-VRC01-*N*. (**A**) log–log dose–response relationships summarized in Table 1. (**B**) Combination index (*CI*) plot for TDF-FTC-VRC01-*N.* Open circles correspond to datapoints from separate experiment. *CI* > 1 antagonism; *CI* = 1 (broken line), additive effect; *CI* < 1 synergism. (**C**) Dose-reduction index (DRI) plot for TDF-FTC-VRC01-*N*. The DRI of 1 shown as a broken line represents no dose reduction relative to the drugs evaluated individually. (**D**) Predicted EC_50_–EC_97_ values for TDF-FTC-VRC01-*N*.

The median-effect model based on mass action^24,25^ unifies important biochemical and biophysical equations. It is used for quantitative analysis of the dose–effect relationship in pharmacodynamic studies and is particularly suited for drug combinations. The median-effect data used in our analysis are provided in Appendix D in the Supplementary Information. Using this mathematical approach, dose–effect relationships were transformed from the classical sigmoidal curves shown in Fig. 2 to straight lines (Fig. 3A). The slopes of these lines, *m*, (Table 1) are a measure of the steepness of the dose–response relationship and, therefore, provide a convenient representation of drug potency (the larger the *m*-value, the more potent the agent) for direct comparison. Figure 3A shows the linearized dose–response relationships for the individual drugs as well as the triple combination. The median-effects principle also allows the biological effect of drug combinations to be studied empirically using a combination index (*CI*) plot (Fig. 3B). If the effect of the drug combination is simply additive, *CI* = 1. Antagonism is defined by *CI* > 1 and synergism by *CI* < 1. Above the ED_50_ (fraction affected, *F*_*a*_, > 0.5) the *CI* of the TDF-FTC-VRC01-*N* combination was found to be synergistic. The *CI* index values for the triple combination at the ED_95_ and ED_97_ concentrations were 0.71 and 0.66, respectively, and approached 0.4 at *F*_*a*_ = 1.0. Synergism leads to a potential concomitant dose reduction of the drug regimen that may, in turn, hold safety and cost benefits. The median-effect model was used to calculate the dose-reduction index (DRI) as a function of *F*_*a*_ (Fig. 3C) independently for all three agents. Finally, the linear dose–response analysis was used to calculate the dose of individual agents in the triple combination required to achieve *F*_*a*_ values (i.e., EDs) informative for HIV-1 prevention (Fig. 3D).

## Discussion

An oral regimen of TDF and FTC (Truvada, Gilead Sciences, Foster City, CA) is FDA-approved for HIV-1 PrEP^28^, so we hypothesized that topical delivery of these ARV agents also would provide protection from vaginal HIV-1 infection. Using this binary combination, we achieved full protection from simian/human immunodeficiency virus (SHIV) infection in the rigorous, repeat low-dose vaginal exposure model using normally cycling female pigtailed macaques^29^ as well as from vaginal and rectal HIV-1 infection in humanized BLT mice^27^. We also showed that topical delivery of the combination from an intravaginal ring (IVR) was safe in a Phase I clinical trial (*N* = 6) and led to drug concentrations in vaginal fluids and tissues exceeding those obtained by highly protective oral dosing, suggesting that efficacy for vaginal HIV-1 PrEP is achievable^30^. The TDF-FTC IVRs also led to unexpectedly high drug concentrations in rectal fluids, indicating that dual-compartment protection HIV-1 infection during both vaginal and receptive anal intercourse may be possible with minimal systemic drug exposure^31^.

Here, we built on the highly promising TDF-FTC foundation and added the broadly neutralizing antibody (bNAb) VRC01 as a third anti-HIV-1 agent. Systemic and topical VRC01 is being investigated clinically as a candidate to prevent sexual HIV-1 transmission^21^, and topical, vaginal administration has shown promise in humanized mice^32,33^ and rhesus macaques^34^. *Nicotiana*-manufactured VRC01 (VRC01-*N*) was used in the current study as this platform has the potential of cost-effectively manufacturing the material at scale without a loss in anti-HIV-1 activity relative to the bNAb produced in HEK cell culture^26^.

There are theoretical advantages for topical HIV-1 PrEP in combining agents with different modes of action, ideally with activity in separate anatomic compartments. Because the activity of VRC01-*N* results from its ability to bind the gp120 surface unit in HIV-1, the bNAb likely needs to be present predominantly in the vaginal fluids. In contrast, the two small molecule ARV drugs, TDF and FTC, inhibit viral reverse transcriptase and topical delivery primarily is targeted at immune cells that support HIV-1 replication in the vaginal tissues. The humanized BLT mouse studies described here were designed to determine empirically if synergy between the agents exists in preventing vaginal HIV-1 infection using the combination index (*CI*) method based on the median-effect principle of the mass-action law^24,25^.

Here, we used 8–10 mice per group and five dosing groups per study (Appendices A–C under Supplementary Information) to generate the dose–response datasets required for the analysis. A constant ratio of drug concentrations in the combinations is used for all groups. The ratio is based on the ED_50_ of the single drug. The dose is defined by *n*·ED_50_ in the fixed combination, where *n* typically spans 0.25–4^24,25^. No control or placebo groups are needed with this approach as the 0% (all animals infected) and 100% (no animals infected) efficacy endpoints are bracketed by the appropriate drug dose combinations administered to each group with the same viral inoculum. This is a significant advantage over the Kaplan–Meier approach used traditionally. The Kaplan–Meier design is a non-parametric survival technique that compares the median survival time of different groups. Our goal was to characterize the synergistic potential of drug combinations on preventing vaginal HIV-1 infection of the mice. The median-effect method allowed us to assess synergy and survival (i.e., efficacy) with fewer mice than a factorial design based on Kaplan–Meier. We would require approximately 15 mice per group in a factorial design to have similar power using the log-rank test.

Measuring synergism or antagonism for drug combinations using the median-effect principle requires a priori knowledge of the potency and the shape of the dose–effect curve for each drug. The dose–effect parameters of each drug (Figs. 2A, 3A, Table 1) were first measured individually, and then applied to design the combination experiments (Figs. 2B, 3A) used to calculate the *CI* plot (Fig. 3B). The slope parameter, *m*, is analogous to the Hill coefficient and describes the sigmoidicity of the dose–effect curve (Table 1). The *m*-values from the TDF and FTC single drug experiments are lower than reported previously^27^ as less extreme boundary conditions were used here to minimize biases in the current analysis (see “Methods^”^, ^“^Data analysis”). *F*_*a*_ values of 0.0025 and 0.99 used here at 0 and 100% efficacy, respectively, rather than 0.000001 and 0.9999. The high *m*-value measured for the triple combination (5.62, Table 1) reflects the steepness of the dose–response curve as illustrated by the median-effect plots (Fig. 3A) and results in a relatively flat change in concentration between the ED_50_ and ED_97_ values (Fig. 3D).

We previously demonstrated that the nucleotide/nucleoside reverse-transcriptase inhibitors (NRTIs) TDF (analog of adenosine 5′-monophosphate) and FTC (analog of cytidine) were mildly antagonistic in preventing vaginal and rectal HIV-1 acquisition in the BTL mouse model^27^. Both ARV drugs are substrates for the reverse transcriptase enzyme and, therefore their antagonistic effect was not entirely surprising^35^. However, when VRC01-*N* was added to the combination, a steep reduction in *CI* was observed above *F*_*a*_ values of 0.5—tending to 0.4 as *F*_*a*_ approached 1.0 (Fig. 3B)—demonstrating a high degree of synergy between the agents. This is the first example of synergism in the prevention of HIV-1 infection in vivo.

One of the primary motivations in targeting a synergistic drug combination for vaginal HIV-1 PrEP was to reduce the dose, thereby minimizing potential toxicity while maximizing efficacy. The concept of the dose-reduction index (DRI) was formally introduced by Chou and Talalay^24^ and is a measure of how much the dose of each drug in a synergistic combination can be reduced for a given effect level compared with the doses of each drug alone. A high DRI of fivefold, or more, was obtained for TDF and VRC01-*N* as *F*_*a*_ approached 1.0, while the DRI for FTC was closer to 3 (Fig. 3C).

The results from the current study suggest that the TDF-FTC-VRC01-*N* combination holds significant potential for effective vaginal HIV-1 PrEP. The agents can be administered from on-demand formulations, such as rapidly disintegrating vaginal tablets, or via long-acting delivery from IVRs. These modalities merit further preclinical investigation, with the goal of transitioning lead candidates into clinical trials.

## Methods

### Materials

Tenofovir disoproxil fumarate (TDF) and emtricitabine (FTC) were purchased from Macleods Pharmaceuticals LTD (Umbergaon, Gujarat, India). VRC01-*N* (Batch 08SD51) kindly was provided by Mapp Biopharmaceutical, Inc. (San Diego, CA) as a spray-dried powder formulation containing the active ingredient at ca. 50% w/w^34^. A VRC01 reference standard (500 µg of purified antibody at 1 mg mL^−1^ in PBS, pH 7.2; catalog number 12033, lot number 160229) produced in a HEK 293-6E expression system was obtained from the NIH AIDS Reagent Program (Germantown, MD). All other reagents were obtained from Sigma-Aldrich (St. Louis, MO), unless otherwise noted.

### Characterization of VRC01-*N*

SDS-PAGE gels were run as follows. A predetermined amount of VRC01-*N* was electrophoresed on a Novex NuPAGE 4–12% Bis–Tris protein gel (NP0321BOX, ThermoFisher Scientific, Waltham, MA) with MES SDS Running buffer (NP0002, ThermoFisher Scientific) using an XCell SureLock Electrophoresis System (EI0002, ThermoFisher Scientific) and a Novex Sharp Pre-Stained Protein Standard (LC5800, ThermoFisher Scientific). The resolved proteins were stained with SimplyBlue Safe Stain (LC6065, Thermo Fisher Scientific). Detection was performed using an Odyssey Fc imaging system (LI-COR, Lincoln, NE).

Western blots were carried out as follows. A predetermined amount of VRC01-*N* was electrophoresed on a Novex NuPAGE 4–12% Bis–Tris protein gel (NP0321BOX, ThermoFisher Scientific) using an XCell SureLock Electrophoresis System (EI0002, ThermoFisher Scientific) and a Novex Sharp Pre-Stained Protein Standard (LC5800, ThermoFisher Scientific). The resolved proteins were transferred onto a nitrocellulose membrane with iBlot 2 Dry Blotting System (IB21001, ThermoFisher Scientific). The membrane was probed with the relevant primary (I2136, Sigma-Aldrich) and secondary (A-11058, Thermo Fisher Scientific) antibodies following blocking with 5% skimmed milk. Detection was performed using an Odyssey Fc imaging system (LI-COR).

VRC01-*N* gp120 binding activity was analyzed by ELISA as follows. The HIV-1 gp120 protein coating antigen (Abcam ab73769, recombinant HIV-1 gp120 protein) and goat anti-human kappa-HRP (detection antibody) was obtained from Southern Biotech (Birmingham, AL). Bovine serum albumin (BSA) was obtained from Sigma-Aldrich. SureBlue TMB peroxidase substrate was obtained from KPL, Inc. (Gaithersburg, MD). All measurements were carried out in triplicate. Sample dilutions at eight concentrations from 75 to 10,000 ng mL^−1^ (as determined by OD_280_) were prepared from the VRC01-*N* and VRC01 samples listed above. ELISA plates were prepared by sequentially incubating in 96-well plates with 100 µL per well coating antigen (1 ng μL^−1^ in 1 × PBS), 200 µL per well of blocking buffer (2% w/v BSA in 1 × PBS), 100 µL of standard or sample, and finally 100 µL of detection antibody diluted 1:5,000 in wash buffer. All blocking and incubation steps were at room temperature for 1 h with gentle agitation of the plate. Plates were washed 3–4 times with wash buffer between each incubation step. Plates were developed by adding 100 µL TMB substrate solution to each well, followed by 50 µL 4*N* H_2_SO_4_ once color development was observed in low concentration dilutions. The absorption at 450 nm (OD_450_) was measured using a SpectraMax Plus384 (Molecular Devices, San Jose, CA) microplate reader.

### Animal care and ethics statement

Mice were maintained and efficacy studies were performed in animal biosafety level 3 facilities at the Department of Animal Resources (DAR), at The Scripps Research Institute. All works were conducted under protocols approved by the Institutional Animal Care and Use Committee at The Scripps Research Institute (Permit Number: 13–0001) in strict accordance with the recommendations in *the Guide for the Care and Use of Laboratory Animals of the National Institutes of Health*. All surgery was performed under sodium pentobarbital anesthesia, and all efforts were made to minimize suffering. Cervical dislocation was used as the method of sacrifice*.* A power calculation was used to determine the sample size (number of mice/group), as described in the “Results” section.

### Generation of humanized BLT mice

Humanized BLT mice were generated as described previously^27,36–39^. In summary, ca. 1-mm^3^ pieces of human fetal liver and thymus tissues (Advanced Bioscience Resources, Alameda, CA) were implanted under the kidney capsule in 6- to 8-week-old female NSG mice (Jackson Laboratories, Ellsworth, ME) bred at The Scripps Research Institute. Each cohort was produced with tissues from a single donor. CD34^+^ HSPC were purified from autologous fetal liver tissue, isolated by magnetic bead selection for CD34^+^ cells (Miltenyi Biotec, San Diego, CA), phenotyped cytometrically^27,36–39^, and cryopreserved until injection (200,000–350,000 CD34^+^ cells) into mice 3 weeks after Thy/Liv implantation. Human reconstitution in peripheral blood was verified by flow cytometry as described^27,36–39^. The gating strategy used to determine the degree of humanization has been described elsewhere^40^. Mice with an average > 60% of human CD45^+^ cells were selected to ensure successful HIV-1 infection.

### Vaginal exposure of humanized BLT Mice to HIV-1

Vaginal drug dosing was carried out as described previously^27^. Four sets of infection studies were performed. The first used an exploratory dose (VRC01-*N*: 50 µg mL^−1^, 318 nM; TDF-FTC-VRC01-*N*: TDF, 4,000 nM; FTC, 80 nM; VRC01-*N*, 318 nM) to define the dose range for EC_50_ determination. The second used a narrowed range based on results from the first study set (VRC01-*N*: 199–3,185 nM), as detailed under Appendix A of the Supplementary Information. The third set used a series of five combination doses of TDF, FTC, and VRC01-*N* in a 16:1:3.2 molar ratio [TDF + FTC + VRC01-*N* (all in nM): 1,000 + 63 + 199; 2,000 + 125 + 398; 4,000 + 250 + 796; 8,000 + 500 + 1,592; 16,000 + 1,000 + 3,185), as detailed under Appendix B of the Supplementary Information. The fourth set used a series of five combination doses of TDF, FTC, and VRC01-*N* in a 16:1:3.2 molar ratio [TDF + FTC + VRC01-*N* (all in nM): 500 + 31 + 100; 1,250 + 78 + 299; 1,500 + 94 + 299; 1,750 + 109 + 347; 2,000 + 125 + 398), as detailed under Appendix C of the Supplementary Information.

Each set of concentrations was used for vaginal challenges following the experimental design outlined in Fig. 1. Stocks of HIV-1 JR-CSF were prepared as previously described^36,37^ and standardized by p24 ELISA using the Alliance HIV-1 P24 ANTIGEN ELISA Kit (96 Test) (Perkin Elmer, Waltham, MA), according to the manufacturer’s instructions. Prior to inoculation, mice were anesthetized with isoflurane. Aliquots (5 μL) of drug solutions in PBS were applied vaginally through a pipet tip. The rear half of the mouse remained elevated during the procedure to reduce chance of back-flow from the vaginal cavity during the recovery. Ten to fifteen minutes post-drug application, mice were vaginally challenged with HIV-1 (5 µL, 200 ng of p24). This inoculum is a standard high viral load for successful vaginal infection (1 ng of p24 corresponds to ca. 10 infectious units). Methods used for the atraumatic vaginal HIV-1 challenge are described elsewhere^38,41–44^.

### Analysis of HIV-1 infection of humanized BLT mice

Infection of BLT mice was monitored by quantifying HIV RNA concentrations in peripheral blood (plasma) at weeks 1, 2, 3, 6 and 12 (Fig. 1) using one-step reverse transcriptase qPCR (Applied Biosystems custom TaqMan Assays-by-Design, ThermoFisher Scientific) according to the manufacturer’s instructions. Primers were 5-CATGTTTTCAGCATTATCAGAAGGA-3 and 5-TGCTTGATGTCCCCCCACT-3, and MGB-probe 5-FAM-CCACCCCACAAGATTTAAACACCATGCTAA-Q-3, where FAM is 6-carboxyfluorescein^38,41–44^. The assay sensitivity was of 400 RNA copies per mL.

### Data analysis

Analytic simulations of dose–response curves using the median-effect principle and mass-action law, and its combination index theorem^24,25^ were carried out using CompuSyn^45^. *F*_*a*_ values of 0.0025 and 0.99 were used at 0 and 100% efficacy, respectively. Data were analyzed and plotted in GraphPad Prism (version 8.4.2, GraphPad Software, Inc., La Jolla, CA).

## Supplementary information

Supplementary Information.

## Data Availability

The data underlying Figs. 2 and 3 are available in the associated source data file included in the Supplementary Information. All other data supporting the findings of this manuscript are available from the corresponding authors (MMB and PAG) upon reasonable request.
