# Supplementary material for: Highly synergistic drug combination prevents vaginal HIV infection in humanized mice
Source: Sci Rep. 2020 Aug 3;10:12995. doi: 10.1038/s41598-020-69937-5 (PMC7400648; doi:10.1038/s41598-020-69937-5)
Supplement: Supplementary file 1 — Supplementary Information. [file 41598_2020_69937_MOESM1_ESM.pdf]

Highly Synergistic Drug Combination Prevents Vaginal HIV Infection in Humanized Mice

Marc M. Baum[1*], Christina M. Ramirez[2], John A. Moss[1], Manjula Gunawardana[1], Michael Bobardt[3], Philippe A. Gallay[3*]

[1]Department of Chemistry, Oak Crest Institute of Science, 128-132 W. Chestnut Ave., Monrovia, CA, USA

[2]University of California, Los Angeles (UCLA) Fielding School of Public Health, 650 Charles E. Young Dr. South, 16-035 Center for Health Sciences, Los Angeles, CA, USA

[3]Department of Immunology & Microbiology, The Scripps Research Institute, 10550 North Torrey Pines Road, La Jolla, CA, USA

[*]email: m.baum@oak-crest.org; gallay@scripps.edu

Supplementary Information

The Supplementary Information includes the following data:

Figure S1: VRC01-*N* SDS-PAGE and Western blot images.

Appendix A: BLT mouse efficacy data from HIV vaginal challenge studies following vaginal administration of VRC01-*N*.

Appendix B: BLT mouse efficacy data from HIV vaginal challenge studies following vaginal administration of VRC01-*N*, TDF, FTC (broad dosing range).

Appendix C: BLT mouse efficacy data from HIV vaginal challenge studies following vaginal administration of VRC01-*N*, TDF, FTC (narrow dosing range).

Appendix D: Median-effect data used in the analysis.

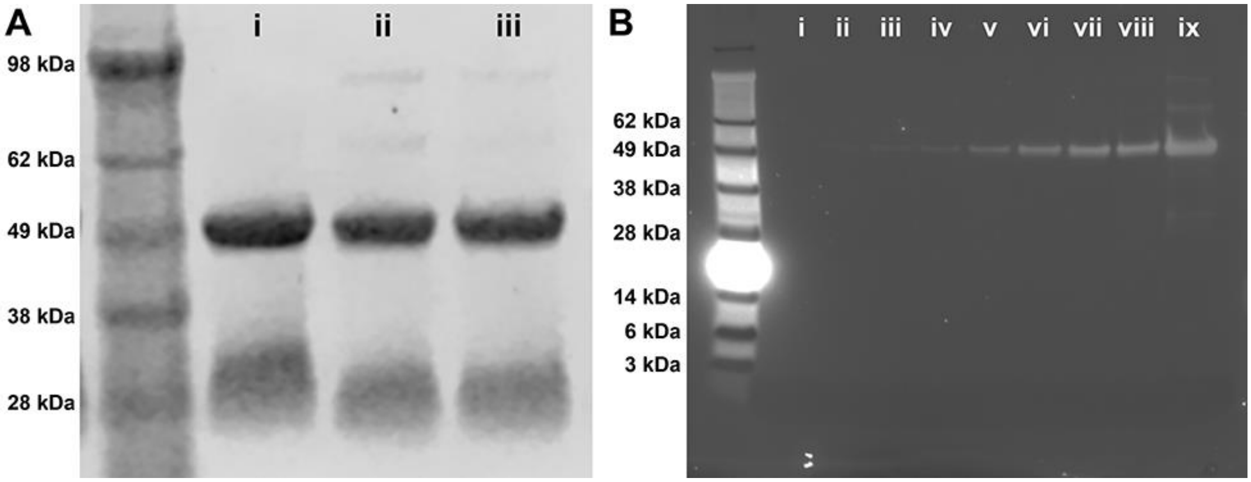

Figure S1. PAGE and Western blot analysis of VRC01. **A**: Coomassie-stained SDS-PAGE gel showing VRC01-*N*. **i**: NIH AIDS Reagent Program VRC01 reference material, 0.60 µg well^{-1}; **ii**: VRC01-*N* produced in *Nicotiana* spp. (2014), this study, 0.33 µg well^{-1}; **iii**: VRC01-*N* produced in *Nicotiana* spp. (2018), 0.31 µg well^{-1}. **B**: Western blot of VRC01-*N* (this study) over a 0.01-5 µg well^{-1} range. **i**: 0.01 µg; **ii**: 0.05 µg; **iii**: 0.075 µg; **iv**: 0.1 µg; **v**: 0.25 µg; **vi**: 0.5 µg; **vii**: 0.75 µg; **viii**: 1.0 µg; **ix**: 5.0 µg.

Appendix A: BLT mouse efficacy data from HIV vaginal challenge studies following vaginal administration of VRC01-*N*.

Mice	Human CD45+ cells (% +/- S.D.)	Human T cells (% +/- S.D.)	HIV-1 positive mice	Week 1	Week 2	Week 3	Week 6	Week 12	VRC01	% of Protection
1	66.2 +/- 0.3	79.2 +/- 1	N	0	0	0	0	0		
2	69.9 +/- 0.5	81.2 +/- 0.9	N	0	0	0	0	0		
3	70.3 +/- 0.5	77.3 +/- 1.1	N	0	0	0	0	0		
4	69.2 +/- 0.4	77.9 +/- 0.7	N	0	0	0	0	0	1 mg/mL	100%
5	66.9+/- 0.5	78.6 +/- 1.2	N	0	0	0	0	0		
6	67.5 +/- 0.5	77.6 +/- 0.8	N	0	0	0	0	0		
7	70.1 +/- 0.4	80.1 +/- 0.7	N	0	0	0	0	0		
8	66.9 +/- 0.5	81.5 +/- 1.1	N	0	0	0	0	0		

Mice	Human CD45+ cells (% +/- S.D.)	Human T cells (% +/- S.D.)	HIV-1 positive mice	Week 1	Week 2	Week 3	Week 6	Week 12	VRC01	% of Protection
1	69.5 +/- 0.8	76.9 +/- 1.2	N	0	0	0	0	0		
2	67.9 +/- 0.7	78.2 +/- 0.8	N	0	0	0	0	0		
3	66.4 +/- 0.8	80.1 +/- 1.1	N	0	0	0	0	0		
4	70.1 +/- 0.5	78.6 +/- 1.3	N	0	0	0	0	0	0.5 mg/mL	100%
5	69.5 +/- 0.6	79.7 +/- 1.4	N	0	0	0	0	0		
6	69.3 +/- 0.7	79.4 +/- 1.3	N	0	0	0	0	0		
7	65.6 +/- 0.5	80.5 +/- 1.5	N	0	0	0	0	0		
8	69.1 +/- 0.7	78.7 +/- 1	N	0	0	0	0	0		

Mice	Human CD45+ cells (% +/- S.D.)	Human T cells (% +/- S.D.)	HIV-1 positive mice	Week 1	Week 2	Week 3	Week 6	Week 12	VRC01	% of Protection
1	68.8 +/- 1.1	76.7 +/- 1.4	N	0	0	0	0	0		
2	66.8 +/- 0.9	78.4 +/- 1.2	N	0	0	0	0	0		
3	70 +/- 1	75.6 +/- 1.5	Y	0.9 +/- 0.2	1.1 +/- 0.2	1.2 +/- 0.3	1.9 +/- 0.4	1.8 +/- 0.3		
4	69.2 +/- 0.9	80.2 +/- 1.3	N	0	0	0	0	0	0.25 mg/mL	50%
5	67.5 +/- 0.7	79.1 +/- 1.1	Y	0.7 +/- 0.2	1 +/- 0.3	1.3 +/- 0.4	2.1 +/- 0.8	1.9 +/- 0.7		
6	64.9 +/- 0.9	77.8 +/- 1.3	Y	0.9 +/- 0.3	1.2 +/- 0.3	1.4 +/- 0.6	1.8 +/- 0.5	2 +/- 0.6		
7	70.1 +/- 1.2	79.3 +/- 1.4	Y	0.8 +/- 0.4	1.1 +/- 0.2	1.5 +/- 0.4	2 +/- 0.6	1.8 +/- 0.2		
8	68.7 +/- 0.9	77.2 +/- 1.1	N	0	0	0	0	0		

Mice	Human CD45+ cells (% +/- S.D.)	Human T cells (% +/- S.D.)	HIV-1 positive mice	Week 1	Week 2	Week 3	Week 6	Week 12	VRC01	% of Protection
1	69.1 +/- 1.1	77.3 +/- 1.1	Y	0.8 +/- 0.2	1.2 +/- 0.3	1.4 +/- 0.3	1.7 +/- 0.3	1.9 +/- 0.4		
2	66.8 +/- 0.7	76.8 +/- 1.3	Y	1 +/- 0.3	1.3 +/- 0.4	1.5 +/- 0.4	1.9 +/- 0.4	1.8 +/- 0.2		
3	70.1 +/- 0.6	78.2 +/- 1.5	Y	0.7 +/- 0.2	0.9 +/- 0.2	1.2 +/- 0.2	1.7 +/- 0.2	1.9 +/- 0.4		
4	67.4 +/- 0.8	80.1 +/- 1.2	Y	0.9 +/- 0.2	1.1 +/- 0.3	1.3 +/- 0.4	2.1 +/- 0.4	1.9 +/- 0.3	0.125 mg/mL	13%
5	65.7 +/- 0.6	76.4 +/- 0.7	Y	0.8 +/- 0.1	1 +/- 0.2	1.2 +/- 0.3	1.9 +/- 0.3	1.9 +/- 0.5		
6	68.3 +/- 0.3	79.3 +/- 1.3	N	0	0	0	0	0		
7	67.6 +/- 0.4	78.4 +/- 0.7	Y	0.9 +/- 0.3	1.2 +/- 0.2	1.4 +/- 0.1	2.2 +/- 0.4	2 +/- 0.3		
8	70.1 +/- 0.5	77.1 +/- 1.4	Y	0.8 +/- 0.2	1 +/- 0.3	1.2 +/- 0.3	1.8 +/- 0.3	1.9 +/- 0.4		

Mice	Human CD45+ cells (% +/- S.D.)	Human T cells (% +/- S.D.)	HIV-1 positive mice	Week 1	Week 2	Week 3	Week 6	Week 12	VRC01	% of Protection
1	68.2 +/- 1.0	76.9 +/- 1.3	Y	0.6 +/- 0.2	0.8 +/- 0.2	1.2 +/- 0.3	1.6 +/- 0.3	1.9 +/- 0.4		
2	66.9 +/- 0.7	78.1 +/- 1.1	Y	0.9 +/- 0.3	1.1 +/- 0.1	1.4 +/- 0.4	1.8 +/- 0.4	2.0 +/- 0.3		
3	70.3 +/- 0.4	75.4 +/- 1.2	Y	1.0 +/- 0.2	1.2 +/- 0.3	1.5 +/- 0.2	1.9 +/- 0.3	2.2 +/- 0.4		
4	67.7 +/- 1.2	78.2 +/- 1.1	Y	0.8 +/- 0.2	1.0 +/- 0.2	1.3 +/- 0.2	1.7 +/- 0.2	1.9 +/- 0.3	0.0625 mg/mL	0%
5	65.7 +/- 0.6	81.2 +/- 0.9	Y	0.9 +/- 0.2	1.2 +/- 0.2	1.4 +/- 0.4	2.1 +/- 0.4	1.8 +/- 0.4		
6	67.1 +/- 0.5	77.6 +/- 1.2	Y	0.7 +/- 0.1	0.9 +/- 0.2	1.2 +/- 0.3	1.7 +/- 0.3	1.8 +/- 0.3		
7	68.7 +/- 0.4	78.1 +/- 0.7	Y	1.1 +/- 0.3	1.3 +/- 0.3	1.5 +/- 0.4	1.9 +/- 0.2	2.1 +/- 0.4		
8	70.5 +/- 0.6	76.1 +/- 1.1	Y	0.8 +/- 0.1	1.1 +/- 0.2	1.3 +/- 0.3	2.0 +/- 0.4	1.9 +/- 0.3		

Plasma viral RNA (10^5 copies/mL)

NOTE: VRC01-N active makes up 50% of the mass indicated above; the remainder consists of excipients

Appendix B: BLT mouse efficacy data from HIV vaginal challenge studies following vaginal administration of VRC01-*N,* TDF, FTC (broad dosing range).

Mice	Human CD45+ cells (% +/- S.D.)	Human T cells (% +/- S.D.)	HIV-1 positive mice	Week 1	Week 2	Week 3	Week 6	Week 12	TDF/FTC/VRC01	% of Protection
1	70.2 +/- 0.3	75.5 +/- 1.0	N	0	0	0	0	0		
2	68.5 +/- 0.4	78.4 +/- 0.8	Y	0.8 +/- 0.1	1.2 +/- 0.4	1.5 +/- 0.3	1.9 +/- 0.3	1.9 +/- 0.2		
3	69.1 +/- 0.3	75.9 +/- 0.6	Y	0.7 +/- 0.2	1.1 +/- 0.2	1.4 +/- 0.2	2.0 +/- 0.2	2.1 +/- 0.3		
4	68.4 +/- 0.5	75.6 +/- 0.9	Y	0.5 +/- 0.1	0.9 +/- 0.1	1.6 +/- 0.3	2.2 +/- 0.4	2.1 +/- 0.1		
5	71.2+/- 0.6	77.5 +/- 1.2	Y	0.6 +/- 0.2	0.9 +/- 0.2	1.4 +/- 0.2	2.1 +/- 0.5	1.9 +/- 0.4	1 µM TDF + 0.0625 µM FTC + 0.0625 mg/mL VRC01	20%
6	67.9 +/- 0.5	78.9 +/- 0.9	Y	0.9 +/- 0.2	1.2 +/- 0.2	1.3 +/- 0.4	1.9 +/- 0.2	2.0 +/- 0.2		
7	70.2 +/- 0.6	75.7 +/- 0.7	Y	0.7 +/- 0.1	1.1 +/- 0.1	1.6 +/- 0.3	2.1 +/- 0.3	1.9 +/- 0.3		
8	67.8 +/- 0.3	77.3 +/- 1.1	Y	0.6 +/- 0.2	0.9 +/- 0.2	1.4 +/- 0.1	1.9 +/- 0.2	2.0 +/- 0.3		
9	69.3 +/- 0.6	76.8 +/- 0.7	N	0	0	0	0	0		
10	65.8 +/- 0.3	78.5 +/- 0.9	Y	0.8 +/- 0.2	1.3 +/- 0.2	1.6 +/- 0.2	2.2 +/- 0.3	2.1 +/- 0.2		

Mice	Human CD45+ cells (% +/- S.D.)	Human T cells (% +/- S.D.)	HIV-1 positive mice	Week 1	Week 2	Week 3	Week 6	Week 12		% of Protection
1	71.2 +/- 0.4	76.6 +/- 1.2	N	0	0	0	0	0		
2	69.5 +/- 0.7	74.9 +/- 0.8	N	0	0	0	0	0		
3	67.9 +/- 0.5	78.2 +/- 1.1	N	0	0	0	0	0		
4	70.1 +/- 0.7	78.8 +/- 0.7	N	0	0	0	0	0		
5	68.5 +/- 0.5	76.4 +/- 1.1	N	0	0	0	0	0	2 µM TDF + 0.125 µM FTC + 0.125 mg/mL VRC01	100%
6	66.9 +/- 0.7	77.4 +/- 1.2	N	0	0	0	0	0		
7	69.9 +/- 0.4	77.3 +/- 1.1	N	0	0	0	0	0		
8	68.5 +/- 0.6	75.8 +/- 0.6	N	0	0	0	0	0		
9	69.8 +/- 0.6	81.2 +/- 1.2	N	0	0	0	0	0		
10	68.4 +/- 0.5	78.3 +/- 0.9	N	0	0	0	0	0		

Mice	Human CD45+ cells (% +/- S.D.)	Human T cells (% +/- S.D.)	HIV-1 positive mice	Week 1	Week 2	Week 3	Week 6	Week 12		% of Protection
1	69.3 +/- 0.6	77.5 +/- 1.2	N	0	0	0	0	0		
2	67.6 +/- 0.9	78.5 +/- 1.2	N	0	0	0	0	0		
3	70.1 +/- 0.5	74.5 +/- 0.8	N	0	0	0	0	0		
4	69.1 +/- 0.8	73.8 +/- 0.6	N	0	0	0	0	0		
5	65.4 +/- 1.1	77.2 +/- 0.9	N	0	0	0	0	0	4 µM TDF + 0.25 µM FTC + 0.25 mg/mL VRC01	100%
6	68.6 +/- 0.5	81.3 +/- 1.3	N	0	0	0	0	0		
7	65.6 +/- 0.8	79.4 +/- 0.8	N	0	0	0	0	0		
8	68.5 +/- 0.7	77.2 +/- 1.1	N	0	0	0	0	0		
9	71.2 +/- 0.6	75.6 +/- 1.2	N	0	0	0	0	0		
10	66.5 +/- 0.7	77.4 +/- 1.0	N	0	0	0	0	0		

Mice	Human CD45+ cells (% +/- S.D.)	Human T cells (% +/- S.D.)	HIV-1 positive mice	Week 1	Week 2	Week 3	Week 6	Week 12		% of Protection
1	69.3 +/- 0.9	76.5 +/- 1.1	N	0	0	0	0	0		
2	65.8 +/- 0.6	74.9 +/- 1.2	N	0	0	0	0	0		
3	70.1 +/- 1.1	75.5 +/- 0.9	N	0	0	0	0	0		
4	66.8 +/- 0.8	77.6 +/- 1.1	N	0	0	0	0	0		
5	66.4 +/- 0.5	79.2 +/- 0.9	N	0	0	0	0	0	8 µM TDF + 0.5 µM FTC + 0.5 mg/mL VRC01	100%
6	68.3 +/- 0.9	81.0 +/- 1.3	N	0	0	0	0	0		
7	66.5 +/- 0.6	76.5 +/- 0.7	N	0	0	0	0	0		
8	71.2 +/- 0.7	79.2 +/- 1.1	N	0	0	0	0	0		
9	69.1 +/- 1.2	75.6 +/- 1.3	N	0	0	0	0	0		
10	66.4 +/- 0.9	77.5 +/- 1.2	N	0	0	0	0	0		

Mice	Human CD45+ cells (% +/- S.D.)	Human T cells (% +/- S.D.)	HIV-1 positive mice	Week 1	Week 2	Week 3	Week 6	Week 12		% of Protection
1	69.2 +/- 1.0	76.1 +/- 1.1	N	0	0	0	0	0		
2	66.5 +/- 0.8	74.1 +/- 1.2	N	0	0	0	0	0		
3	68.4 +/- 0.6	75.4 +/- 1.1	N	0	0	0	0	0		
4	67.3 +/- 0.8	78.5 +/- 1.3	N	0	0	0	0	0		
5	67.4 +/- 0.9	74.7 +/- 0.9	N	0	0	0	0	0	16 µM TDF + 1 µM FTC + 1 mg/mL VRC01	100%
6	69.4 +/- 0.5	79.3 +/- 1.2	N	0	0	0	0	0		
7	66.4 +/- 0.8	75.4 +/- 0.8	N	0	0	0	0	0		
8	65.6 +/- 0.6	75.4 +/- 1.1	N	0	0	0	0	0		
9	69.4 +/- 0.9	76.3 +/- 1.3	N	0	0	0	0	0		
10	66.5 +/- 0.7	75.9 +/- 1.2	N	0	0	0	0	0		

Plasma viral RNA (10^5 copies/mL)

NOTE: VRC01-N active makes up 50% of the mass indicated above; the remainder consists of excipients

Appendix C: BLT mouse efficacy data from HIV vaginal challenge studies following vaginal administration of VRC01-*N,* TDF, FTC (narrow dosing range).

Mice	Human CD45+ cells (% +/- S.D.)	Human T cells (% +/- S.D.)	HIV-1 positive mice	Week 1	Week 2	Week 3	Week 6	Week 12		% of Protection
1	68.4 +/- 0.6	73.8 +/- 2.4	N	0	0	0	0	0		
2	69.9 +/- 0.4	71.1 +/- 1.7	N	0	0	0	0	0		
3	70.1 +/- 0.8	75.5 +/- 2	N	0	0	0	0	0		
4	67.6 +/- 1.2	76.1 +/- 1.5	N	0	0	0	0	0		
5	69.6 +/- 0.9	73.8 +/- 2.4	N	0	0	0	0	0	2 µM TDF + 0.125 µM FTC + 0.125 mg/mL VRC01	100%
6	65.8 +/- 1.1	75.2 +/- 2.2	N	0	0	0	0	0		
7	69.3 +/- 0.7	76.1 +/- 1.5	N	0	0	0	0	0		
8	66.4 +/- 1	73.7 +/- 1.9	N	0	0	0	0	0		
9	69.2 +/- 1.2	76.1 +/- 2.3	N	0	0	0	0	0		
10	65.6 +/- 1.4	74.9 +/- 1.7	N	0	0	0	0	0		

Mice	Human CD45+ cells (% +/- S.D.)	Human T cells (% +/- S.D.)	HIV-1 positive mice	Week 1	Week 2	Week 3	Week 6	Week 12		% of Protection
1	66.4 +/- 1.3	73.5 +/- 2.3	N	0	0	0	0	0		
2	63.8 +/- 2.4	76.1 +/- 1.8	N	0	0	0	0	0		
3	65.1 +/- 1.8	72.6 +/- 2.5	Y	0.7 +/- 0.2	1.2 +/- 0.3	1.5 +/- 0.2	2.1 +/- 0.3	2.2 +/- 0.3		
4	65.7 +/- 1.5	74.2 +/- 1.9	N	0	0	0	0	0		
5	64.6 +/- 2.2	73.8 +/- 1.6	N	0	0	0	0	0	1.75 µM TDF + 0.1094 µM FTC + 0.109 mg/mL VRC01	90%
6	63.8 +/- 1.9	77.2 +/- 2.1	N	0	0	0	0	0		
7	64.6 +/- 1.3	74.2 +/- 1.6	N	0	0	0	0	0		
8	66.7 +/- 1.3	76.2 +/- 2.2	N	0	0	0	0	0		
9	66.8 +/- 2.1	74.6 +/- 1.8	N	0	0	0	0	0		
10	63.7 +/- 2.2	73.7 +/- 2	N	0	0	0	0	0		

Mice	Human CD45+ cells (% +/- S.D.)	Human T cells (% +/- S.D.)	HIV-1 positive mice	Week 1	Week 2	Week 3	Week 6	Week 12		% of Protection
1	65.2 +/- 1.1	74.2 +/- 1.5	N	0	0	0	0	0		
2	62.9 +/- 1.4	77.1 +/- 2.1	N	0	0	0	0	0		
3	63.6 +/- 1.5	73.7 +/- 2.3	Y	0.6 +/- 0.1	1.1 +/- 0.2	1.6 +/- 0.4	2.3 +/- 0.2	2.4 +/- 0.2		
4	66.1 +/- 2.1	77.1 +/- 2.4	N	0	0	0	0	0		
5	62.9 +/- 1.6	75.2 +/- 1.8	N	0	0	0	0	0	1.5 µM TDF + 0.0938 µM FTC + 0.0938 mg/mL VRC01	70%
6	64.4 +/- 2	75.3 +/- 1.6	Y	0.7 +/- 0.2	1.3 +/- 0.3	1.8 +/- 0.3	2.2 +/- 0.3	2.3 +/- 0.4		
7	65.2 +/- 1.7	75.1 +/- 2.2	N	0	0	0	0	0		
8	68.3 +/- 1.3	77.4 +/- 1.8	Y	0.9 +/- 0.3	1.4 +/- 0.2	1.8 +/- 0.4	2.4 +/- 0.4	2.2 +/- 0.3		
9	63.1 +/- 1.5	75.5 +/- 2.4	N	0	0	0	0	0		
10	65.4 +/- 1.9	73.7 +/- 1.9	N	0	0	0	0	0		

Mice	Human CD45+ cells (% +/- S.D.)	Human T cells (% +/- S.D.)	HIV-1 positive mice	Week 1	Week 2	Week 3	Week 6	Week 12		% of Protection
1	64.4 +/- 2.2	73.4 +/- 2.2	Y	0.6 +/- 0.2	1.0 +/- 0.2	1.4 +/- 0.3	2.1 +/- 0.4	2.3 +/- 0.3		
2	62.9 +/- 1.6	71.3 +/- 1.6	N	0	0	0	0	0		
3	64.6 +/- 2.1	73.3 +/- 1.8	N	0	0	0	0	0		
4	66.2 +/- 1.5	74.2 +/- 1.3	Y	0.7 +/- 0.1	1.3 +/- 0.3	1.7 +/- 0.3	2.4 +/- 0.2	2.2 +/- 0.4		
5	65.1 +/- 1.8	71.8 +/- 1.5	Y	0.5 +/- 0.1	1.2 +/- 0.2	1.5 +/- 0.2	2.3 +/- 0.1	2.4 +/- 0.3	1.25 µM TDF + 0.0781 µM FTC + 0.0781 mg/mL VRC01	40%
6	63.3 +/- 1.5	75.7 +/- 2.1	Y	0.4 +/- 0.2	1.0 +/- 0.3	1.6 +/- 0.3	2.2 +/- 0.3	2.3 +/- 0.1		
7	66.2 +/- 2.3	77.2 +/- 1.6	N	0	0	0	0	0		
8	65.3 +/- 1.9	72.9 +/- 1.7	Y	0.6 +/- 0.1	1.2 +/- 0.3	1.7 +/- 0.4	2.2 +/- 0.4	2.6 +/- 0.2		
9	63.8 +/- 2.1	74.2 +/- 2.1	N	0	0	0	0	0		
10	64.1 +/- 1.7	73.7 +/- 1.9	Y	0.5 +/- 0.2	1.3 +/- 0.4	1.8 +/- 0.3	2.4 +/- 0.3	2.4 +/- 0.6		

Mice	Human CD45+ cells (% +/- S.D.)	Human T cells (% +/- S.D.)	HIV-1 positive mice	Week 1	Week 2	Week 3	Week 6	Week 12		% of Protection
1	63.1 +/- 0.6	72.5 +/- 2.2	Y	0.4 +/- 0.1	1.2 +/- 0.3	1.6 +/- 0.3	2.2 +/- 0.4	2.3 +/- 0.1		
2	64.1 +/- 1.2	70.8 +/- 1.6	Y	0.5 +/- 0.2	0.9 +/- 0.1	1.4 +/- 0.2	1.8 +/- 0.3	2.2 +/- 0.3		
3	62.9 +/- 2.1	75.5 +/- 1.1	Y	0.6 +/- 0.2	1.0 +/- 0.2	1.6 +/- 0.4	2.1 +/- 0.3	2.6 +/- 0.5		
4	65.2 +/- 1.7	76.3 +/- 3.1	Y	0.6 +/- 0.1	1.3 +/- 0.2	1.5 +/- 0.3	2.3 +/- 0.4	2.4 +/- 0.4		
5	63.2 +/- 2.3	73.3 +/- 0.7	Y	0.4 +/- 0.2	0.9 +/- 0.2	1.5 +/- 0.1	2.0 +/- 0.3	2.3 +/- 0.2	0.5 µM TDF + 0.0313 µM FTC + 0.0313 mg/mL VRC01	0%
6	65.3 +/- 3.1	75.5 +/- 1.9	Y	0.5 +/- 0.1	1.1 +/- 0.3	1.5 +/- 0.2	2.2 +/- 0.4	2.6 +/- 0.4		
7	64.4 +/- 2.5	71.6 +/- 2.4	Y	0.5 +/- 0.2	1.3 +/- 0.1	1.7 +/- 0.3	2.1 +/- 0.2	2.3 +/- 0.4		
8	66.1 +/- 1.9	74.8 +/- 1.6	Y	0.6 +/- 0.2	1.2 +/- 0.1	1.7 +/- 0.4	2.5 +/- 0.4	2.4 +/- 0.2		
9	64.4 +/- 2.2	72.7 +/- 0.9	Y	0.3 +/- 0.1	0.9 +/- 0.2	1.3 +/- 0.2	1.9 +/- 0.3	2.2 +/- 0.4		
10	62.8 +/- 3.3	71.5 +/- 1.8	Y	0.4 +/- 0.2	1.0 +/- 0.2	1.7 +/- 0.2	2.4 +/- 0.5	2.4 +/- 0.2		

Plasma viral RNA (10^5 copies/mL)

NOTE: VRC01-N active makes up 50% of the mass indicated above; the remainder consists of excipients

Appendix D: Median-effect data used in the analysis.

Median-Effect Data

VRC01-N, single drug

Log(D) (nM)	Efficacy (%)
3.503	100
3.202	100
2.901	50
2.600	13
2.299	0

TDF-FTC-VRC01-N

log(D) (nM)	Efficacy (%) Study 1	Efficacy (%) Study 2
3.101	20	
3.402	100	
3.703	100	
4.004	100	
4.305	100	
2.800		0
3.198		40
3.277		70
3.344		90
3.402		100

Log(D) (nM)	TDF	FTC	VRC01-N	Triple
3.000	-2.601			
3.301	-0.954			
3.602	-0.176			
4.204	1.996			
4.505	1.996			
1.398		-1.591		
1.699		-0.954		
2.301		0.000		
2.602		0.602		
2.778		1.996		
3.503			1.996	
3.202			1.996	
2.901			0.000	
2.600			-0.845	
2.299			-2.601	
3.101				-0.602
2.800				-2.601
3.198				-0.176
3.277				0.368
3.344				0.954
3.402				1.996

Fa	CI Study 1	CI Study 2
0.0025		2.84
0.2	1.30	
0.4		1.19
0.7		0.96
0.9		0.74
0.99	0.40	
0.999	0.40	
0.9999	0.41	

Fa	DRI TDF	DRI FTC	DRI VRC01-N
0.0025	1.47	0.64	1.68
0.2	3.24	1.64	2.66
0.4	3.56	1.85	2.72
0.7	4.45	2.41	3.10
0.9	5.90	3.34	3.73
0.99			5.94
0.999			5.29
0.9999			4.71
